# Biodegradable Active Food Films: Bibliometric Analysis and Literature Review over the Last Five Years

**DOI:** 10.3390/molecules31132266

**Published:** 2026-06-29

**Authors:** Bianca Șuian, Sonia Amariei, Ancuța Petraru

**Affiliations:** Faculty of Food Engineering, Ștefan cel Mare University of Suceava, 720229 Suceava, Romania; bianca.suian@usv.ro (B.Ș.); ancuta.petraru@fia.usv.ro (A.P.)

**Keywords:** active films, bioactive constituents, biodegradable polymers, sustainable packaging

## Abstract

With the continuous evolution of the food industry, extending the shelf life of products while maintaining quality and safety has become a major challenge, alongside growing environmental concerns related to conventional plastic packaging. This study aims to provide an overview of recent advances in active biodegradable films as sustainable alternatives for food applications. A comprehensive review of the relevant literature was conducted, including bibliometric analysis to identify key research directions, emerging trends, and technological developments in the field. Our findings highlight the growing interest in biodegradable polymers incorporated with active compounds, such as antioxidants and antimicrobial agents, which contribute to delaying degradation processes and preserving food freshness. Additionally, the analysis emphasizes the mechanisms of action of these active substances and the factors influencing the biodegradability of packaging materials. The results also reveal a shift toward environmentally friendly solutions driven by the need to reduce plastic waste and improve sustainability. In conclusion, active biodegradable films represent a promising approach to enhancing food preservation while minimizing environmental impact, although further research is needed to optimize material performance, scalability, and industrial applicability.

## 1. Introduction

The packaging industry is undergoing continuous development, particularly in the food sector, where food and beverages account for the largest share, namely 85% [1]. According to Statista (2025, Statista database, accessed in 2026), the global food-packaging market, which has been valued at approximately 385.4 billion U.S. dollars in recent years, is expected to grow and reach about 512 billion U.S. dollars by 2028. Packaging protects food products against physical, chemical and biological factors at various stages of the supply chain [2]. In particular, protection against external influences such as temperature, humidity, and light makes food packaging essential for maintaining the freshness and integrity of products [3]. In addition to its protective function, packaging also plays a key role in determining product composition and functional performance [4]. Current trends focus on the use of active, biodegradable, and edible packaging systems based on biopolymers [5].

At the same time, in a highly competitive market where marketing is rapidly expanding, the commercial aspect should not be overlooked, as a balance between functionality and design is required [3]. Packaging serves as a communication tool [5] and a means of advertising [4]. Consumers’ acceptance of food products is influenced by the colour and appearance of the packaging materials [6], and they show a preference for transparent or near-transparent systems that allow visualization of the product inside [7,8,9]. However, the use of opaque or less transparent packaging is necessary for products with high sensitivity to light [6,7] in order to limit light penetration and preserve product quality [9].

When considering conventional packaging, the main options include plastic, paper, and glass [3,10], along with metal, cardboard, and aluminum [1,11].The selection is made depending on material properties and specific application requirements [12]. At the European level, the food-packaging industry accounts for approximately 40% of the total plastic market [3,13,14,15]. Most conventional plastics are derived from non-renewable resources and are not biodegradable, and some do not have fully established recycling processes [16]. Due to their non-biodegradable and non-renewable nature, plastics represent a significant environmental concern [4,5,17,18].

**Figure 1:**
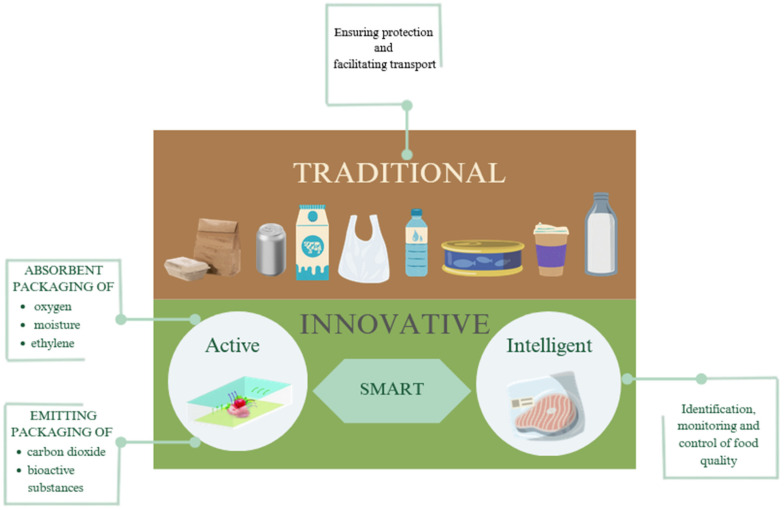
Classification of packaging types in food industry.

The use of biodegradable packaging films actively contributes to the objectives set out in the 2030 Agenda [19], which promotes biodegradable packaging and alternative solutions to conventional plastics, thereby contributing to the reduction in generated waste. Natural sources such as polysaccharides, proteins and lipids [19,20,21] are considered in this context. Another way to achieve this goal is by extending the shelf life of food products [22], which provides a basis for the development of active packaging [23]. Environmental concerns have led to the development of alternative packaging solutions, including packaging made from biodegradable polymers, edible films, and active or intelligent packaging systems [3]. These are based on the incorporation of naturally derived agents [23]. A classification of packaging types used in the food industry is presented in Figure 1.

For these reasons, the present paper aims to provide an update to review the biodegradable active food films used in food packaging, with emphasis on their role in maintaining food quality, supporting preservation, and reducing the environmental impact associated with conventional packaging materials. This review focuses specifically on biodegradable film-forming matrices and active compounds that can provide functional properties, particularly antioxidant and antimicrobial effects, without compromising consumer safety or the acceptability of packaged products.

Although several reviews have addressed biodegradable, edible, antimicrobial, active, or smart food packaging, fewer studies have focused specifically on biodegradable active food films as integrated systems combining biodegradable matrices, active compounds, functional performance, and food-related applicability. Moreover, previous bibliometric studies have examined related topics, including the bibliometric visualization analysis of active packaging systems for food packaging reported by Dirpan et al. [24] for the period 2000–2021. However, that study approached active packaging as a broader research field, whereas the present paper specifically focuses on biodegradable active food films published between 2021 and 2025 and combines a bibliometric analysis of the full dataset with a focused review of selected recent studies. This approach provides an updated perspective on publication trends, research directions, collaborative patterns, commonly used materials and active compounds, and remaining gaps related to performance, real food applications, biodegradability, safety, and scalability.

## 2. Results

In the context of a large volume of scientific literature and its ongoing rapid expansion, detailed data analysis plays an essential role not only in synthesizing information, but also in understanding the geographical distribution of publications in the selected research field [24]. Such analyses provide a comprehensive overview of the scientific landscape and help to identify emerging directions for future research.

Using the Bibliometrix R package (software version 2026.04.0+526) through its Biblioshiny interface and the VOSviewer software (software version 1.6.20), both of which were specifically designed for bibliometric analysis and data visualization, several graphical representations were generated by focusing on key research elements. The obtained results revealed a significant increase in the number of publications during the 2021–2025 period, indicating a growing scientific interest in the development of active biodegradable food-packaging systems. This trend reflects current industrial and environmental demands related to sustainability, efficiency, and innovation.

In addition to providing quantitative insights into publication trends, the bibliometric analysis complements the literature review by identifying the main research themes, influential authors, leading countries, and collaboration networks within the field. This approach enables a more comprehensive understanding of the evolution of scientific knowledge regarding active biodegradable food-packaging systems. Furthermore, the mapping of keywords and research clusters highlights emerging topics and knowledge gaps, offering valuable directions for future investigations and supporting the development of innovative and sustainable packaging solutions.

### 2.1. Bibliometric Analysis

#### 2.1.1. Current Status of Research Performance

The analyzed publications covered the period from 2021 to 2025 and comprised a total of 1801 scientific articles. These documents were examined to provide a comprehensive overview of the research landscape in the field of biodegradable active food packaging. The study involved contributions from 7411 authors across 77 countries, while the average number of citations per document reached 27.7, indicating the significant scientific impact and increasing interest in this research area.

#### 2.1.2. Annual Production of Publications

**Figure 2:**
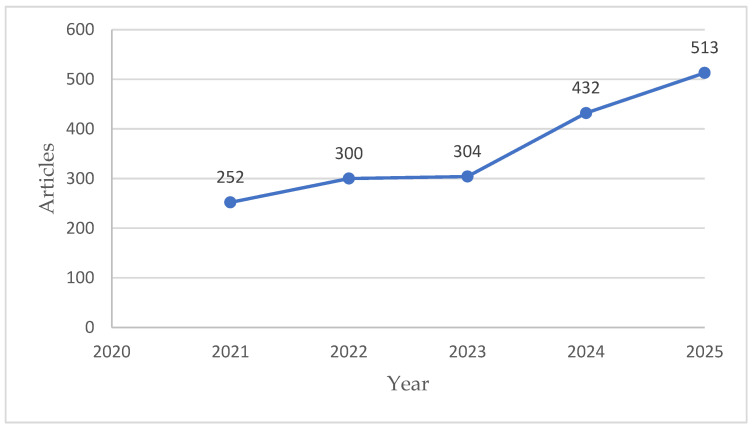
Annual scientific production trends in biodegradable active food films research identified through the Scopus-based bibliometric analysis for the 2021–2025 period.

The evolution of scientific production during the selected period (2021–2025), as illustrated in Figure 2, demonstrates a progressive increase in publication activity.

The number of publications started at 252 articles in 2021 and increased to 302 articles in 2022. Between 2022 and 2023, only a modest increase in four additional publications was observed, possibly reflecting a temporary stabilization phase in research activity or the increasing complexity of studies conducted in the field. However, publication activity expanded considerably from 2024 onward, reaching 432 published articles and peaking at 513 publications in 2025.

#### 2.1.3. Keyword Co-Occurrence and Cluster Analysis

Keyword co-occurrence maps reflect the relationships between the author keywords used in the selected publications. These terms highlight current research trends and contribute to the identification and monitoring of scientific developments within the field [19]. Through the keywords provided by the authors, it is possible to identify major research themes and emerging sub-themes related to biodegradable active food packaging.

**Figure 3:**
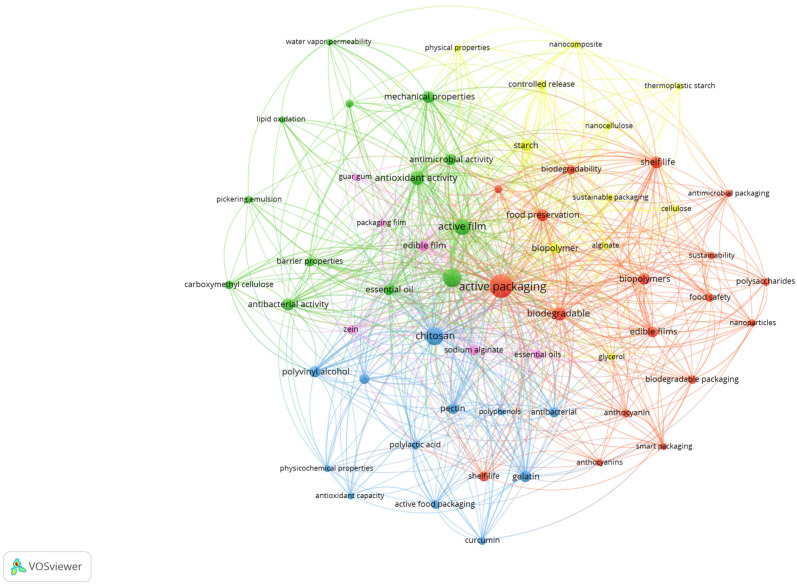
Keyword co-occurrence network identified in the bibliometric analysis of biodegradable active food films research using the Scopus database and the selected search keywords during the 2021–2025 period (VOSviewer visualization). Based on the VOSviewer analysis, five major interconnected clusters were identified and visually represented using different colours (red, green, blue, yellow, and purple). The colour coding highlights the relationships between keywords and their belonging to the same thematic category [19]. The representative themes and keywords associated with each cluster are summarized in Table 1.

In the present bibliometric study, the most frequently used author keywords were visualized as interconnected node networks (Figure 3), allowing a more comprehensive understanding of the conceptual structure of the research field. The visualization was generated using the VOSviewer software, with the minimum number of keyword occurrences set at 15.

**Table 1:** Thematic clusters identified in biodegradable active food-packaging research.

Cluster Colour	Main Research Direction	Representative Keywords
Red	Smart and sustainable food-packaging systems	Active packaging, food packaging, biodegradability, smart packaging, shelf life
Green	Functional properties and bioactive activity	Antimicrobial activity, antioxidant activity, mechanical properties, barrier properties, water vapour permeability
Blue	Biopolymer-based films and materials	Chitosan, gelatin, pectin, sodium alginate, polyvinyl alcohol, starch
Yellow	Advanced biodegradable materials and nanocomposite systems	Starch, cellulose, nanocomposite, controlled release
Purple	Edible films and functional biopolymer additives	Edible film, essential oil, guar gum, sodium alginate, zein

The size of each node reflects the frequency of keyword occurrence, indicating how often a specific term appears within the analyzed publications. Among the most prominent terms identified in the network are “active film,” “antioxidant activity,” “antimicrobial activity,” and “biodegradable,” highlighting the strong scientific interest in sustainable and functional food-packaging systems. Link thickness reflects the strength of co-occurrence relationships between keywords, indicating how frequently specific terms appear together within the same publications.

The red thematic group mainly includes 18 terms associated with active and sustainable food-packaging systems, emphasizing the increasing importance of biodegradable packaging technologies, food preservation, and shelf-life extension applications. The green thematic group is primarily related to the functional and bioactive properties of biodegradable films, highlighting the growing scientific interest in antimicrobial and antioxidant activities, essential oils, and the physicochemical performance of active packaging materials. The blue thematic group focuses on biopolymer-based materials commonly used in the development of biodegradable active films, including chitosan, gelatin, pectin, polyvinyl alcohol, and polylactic acid. The yellow thematic group reflects emerging research directions associated with advanced biodegradable materials and nanocomposite systems, particularly starch-based materials, nanocellulose, thermoplastic starch, and controlled release technologies. The purple thematic group is associated with edible films and functional biopolymer additives, including sodium alginate, zein, guar gum, and essential oils, which are being investigated for their potential applications in sustainable food-packaging systems.

#### 2.1.4. Thematic Analysis

**Figure 4:**
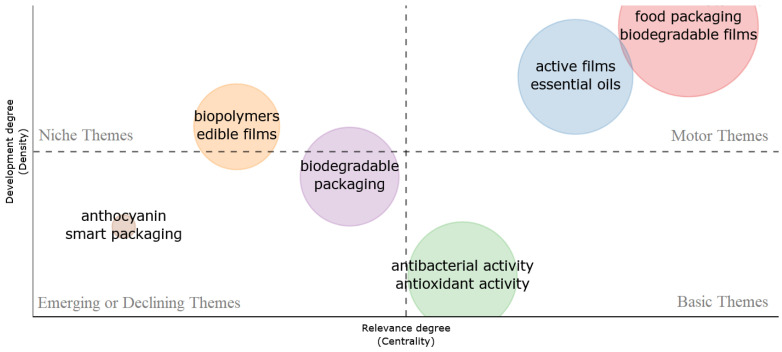
Thematic map of biodegradable active food films research using the Scopus database and the selected search keywords during the 2021–2025 period (Bibliometrix visualization).

Figure 4 presents the thematic map of the selected field based on the keywords used by the authors, with each quadrant representing different characteristics of the mapped themes and concepts. The positioning of the word clusters relative to the two axes, centrality and density, is also significant. Centrality indicates the relevance of the subjects within the overall theme while density provides details regarding their level of development.

The thematic map highlights the research trends, thematic relationships, and potential future directions in the field of biodegradable food packaging. The graphical structure is divided into four quadrants according to density and centrality.

The upper-left quadrant includes the “niche themes,” represented mainly by biopolymers and edible films. These themes are well developed but relatively isolated from the central structure of the field. Their presence reflects the growing interest in natural polymeric matrices and edible coating systems for sustainable packaging applications.

The upper-right quadrant contains the “motor themes,” which are both highly developed and strongly connected to the research field. The dominant cluster is represented by food packaging and biodegradable films, indicating that sustainable packaging systems constitute the core driving direction of current research. Another important motor theme is represented by active films and essential oils. The incorporation of essential oils into active biodegradable films has become increasingly important due to their antimicrobial and antioxidant properties, which improve food preservation and extend shelf life.

The lower-right quadrant represents the “basic themes,” which are fundamental for the development of the domain but still require further investigation. In this area, antibacterial activity and antioxidant activity are the most representative concepts. These properties are essential for the functionality of active packaging systems and are widely investigated in relation to bioactive compounds incorporated into biodegradable matrices.

The lower-left quadrant corresponds to “emerging or declining themes,” represented by anthocyanin and smart packaging. This cluster suggests a growing interest in intelligent packaging systems based on natural pigments capable of monitoring food freshness through colour changes. Although still less developed, these themes show considerable potential for future applications in sustainable food-packaging technologies.

Finally, the theme of biodegradable packaging is positioned close to the centre of the map, indicating its transversal role within the research field. This positioning suggests that biodegradable packaging acts as a connecting topic between multiple research directions and continues to evolve as a major focus in the development of environmentally friendly food-packaging systems.

#### 2.1.5. Distribution of Publications

**Figure 5:**
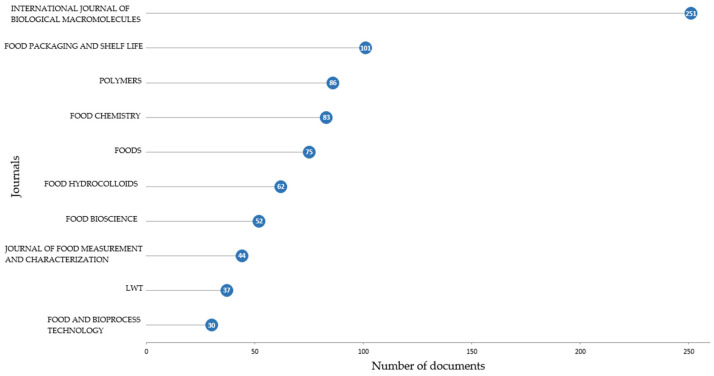
Most relevant journals identified in the bibliometric analysis of biodegradable active food films research using the Scopus database and the selected search keywords during the 2021–2025 period.

The distribution of published articles highlights a considerable diversification of research across multiple disciplinary categories, emphasizing the broad scope and interdisciplinary nature of the topic. The most relevant source identified in the analysis (Figure 5) is the International Journal of Biological Macromolecules (251 documents), followed by Food Packaging and Shelf Life (101 documents), Polymers (86 documents), and Food Chemistry (83 documents).

#### 2.1.6. Authors

With an average of 5.74 co-authors per document, research on biodegradable active food films appears to be highly collaborative and interdisciplinary. This collaborative character is further supported by the international co-authorship rate of 25.21% and by the participation of authors from 77 countries. These results suggest that the field has a broad international dimension and benefits from contributions across different research environments. Moreover, this research area integrates knowledge from multiple scientific domains, such as biodegradable materials, food preservation, and sustainable packaging technologies, thereby requiring a comprehensive and multidisciplinary approach to address the associated challenges.

A significant number of authors contributed to the field of biodegradable active food films during the selected period, 2021–2025. According to the bibliometric results obtained, there are 7411 authors involved in the analyzed research area.

To better characterize author productivity, the top 10 most productive authors were identified based on the number of publications indexed in the dataset and are presented in Table 2.

The most productive authors contributed between 13 and 20 publications, indicating that research activity is distributed across a broad authorship network rather than concentrated around a single author.

Identifying the articles with the highest number of citations is essential to highlight the works that have had the greatest influence on the field of biodegradable active films.

Highly cited papers may indicate a high relevance of some publications within the scientific community, potentially serving as a reference point for the research field. The most cited article in the dataset was “Antimicrobial edible films in food packaging: Current scenario and recent nanotechnological advancements—a review,” which had a total of 374 citations. This article is published in the academic journal “Carbohydrate Polymer Technologies and Application” and underlines the importance of focusing on sustainable sources of materials for the development of food films. The authors Rekha Chawla, S. Sivakumar, and Harsimran Kau highlight the potential of incorporating antimicrobial agents into biopolymer-based edible films [25].

#### 2.1.7. Countries

The bibliometric analysis indicates that there is a strong contribution from Asian countries, particularly China and India, during the 2021–2025 period. However, the results also show relevant contributions from countries in other regions, such as Brazil and several European countries. The geographical distribution of the most active contributing countries is presented in Table 3.

The bibliometric analysis shows that China had the highest level of scientific production, with 3031 country-level contributions linked to authors affiliated with Chinese institutions, followed by Brazil with 1313 contributions and India with 979 contributions. These values indicate country-level bibliometric contribution counts derived from institutional affiliations and do not reflect the number of distinct articles. Therefore, since one publication can include authors from several institutions or countries, the contribution totals may exceed the overall number of articles in the dataset.

China not only exhibited the highest scientific production in the field of active biodegradable films but also ranked first in terms of citation impact, accumulating 15,418 citations during the investigated period.

### 2.2. Literature Review

Conventional plastics are widely used in food packaging because of their low cost, processability, and barrier properties. However, their persistence in the environment, limited recycling rates, and the difficulties associated with recycling food packaging contaminated with organic residues or composed of complex multilayer materials have increased the need for more sustainable alternatives. In this context, biodegradable active food films have attracted growing interest because they can combine environmental advantages with active functions, such as antioxidant or antimicrobial activity, which are aimed at maintaining food quality and extending shelf life.

Because these concepts are sometimes used together in the literature, it is important to distinguish their meanings in the context of this review. Biodegradable packaging refers to materials that can be broken down by micro-organisms under appropriate environmental conditions. Bio-based packaging refers to materials derived fully or partly from renewable biological resources, but these materials are not necessarily biodegradable. Compostable packaging represents a specific category of biodegradable packaging that decomposes under controlled composting conditions. Edible films are materials that can be safely consumed together with the food product. Active packaging interacts with the food or its surrounding environment by releasing or absorbing compounds to extend shelf life or improve food quality and safety. Intelligent packaging monitors the condition of the food or package environment, while smart packaging is generally used as a broader term that may include active and/or intelligent functions [26]. In the present review, these broader packaging categories are discussed only where they directly support the analysis of biodegradable active food films.

#### 2.2.1. Conventional Plastic Packaging and Its Limitation

The most commonly used plastics for food packaging include polyethylene (PE), polyethylene terephthalate (PET), polypropylene (PP), polystyrene (PS), and polyvinyl chloride (PVC), mainly due to their low cost, processability, mechanical strength, and barrier properties [4,27,28].

However, these materials are derived largely from non-renewable resources, persist in the environment, and are associated with limited recycling rates, especially when food packaging is contaminated with organic residues or consists of complex multilayer structures [27,29,30]. Their fragmentation into micro- and nanoplastics has also raised increasing concerns regarding environmental contamination and possible transfer to food-contact systems [31].

These limitations have intensified the search for more sustainable food-packaging solutions. In this context, biodegradable active food films are particularly relevant because they combine biodegradable polymer matrices, often obtained from renewable sources such as polysaccharides, proteins, and lipids, with active compounds that are able to provide antioxidant and/or antimicrobial functions. Such systems can support food preservation while reducing the environmental burden associated with conventional plastic packaging.

#### 2.2.2. The Concept of Biodegradable Packaging

The shift from non-biodegradable plastics to biodegradable packaging has increased the interest in materials obtained from renewable or biodegradable sources [32,33]. In sustainable food packaging, biodegradable polymers are especially relevant because they can reduce long-term environmental persistence while supporting the development of active films for food preservation [34]. Compared to conventional plastics, which often persist in the environment for very long periods, biodegradable polymers may break down more rapidly under suitable environmental or composting conditions [35]. Biodegradable polymers are currently the main point of interest when it comes to the use of materials in the development of biodegradable packaging. However, the percentage of biopolymer-based materials in the global market is still relatively low, although the potential for growth is huge [17].

**Figure 6:**
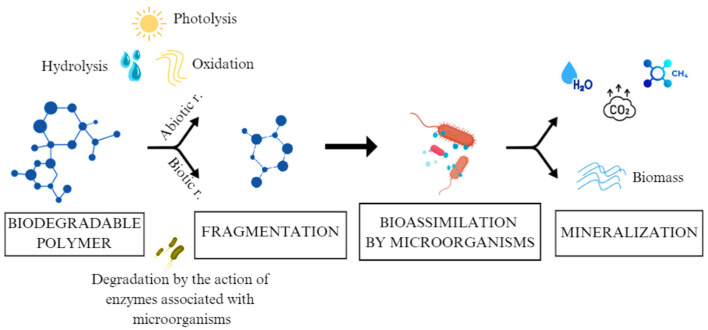
The degradation mechanism of biodegradable polymers through biotic and abiotic reactions [33,37].

The decomposition of biodegradable materials follows a microbial-based pathway influenced by environmental factors such as humidity, temperature, and oxygen, without any negative impact on the surrounding environment [32]. Both the enzymatic hydrolysis of polymer bonds and the process of photolysis are fundamental to the biodegradation process [32]. Degradation involves the fragmentation of polymers into species with lower molecular weight, facilitated by abiotic reactions such as oxidation, photodegradation, hydrolysis and biotic reactions associated with the enzymes produced by micro-organisms growing on the surface of the material, leading to short units of monomers and oligomers [36]. The process ends with the formation of water, carbon dioxide, methane and other reaction products specific to mineralization [37] (Figure 6); these substances are capable of offsetting greenhouse gas emissions [38].

The degradation rate is influenced by several variables, but in terms of sensitivity to the action of microbial enzymes, polymers with shorter chains, more amorphous structures, and lower molecular complexity are favoured [37]. Specific factors such as temperature, light, humidity, pH, UV radiation and exposure to oxygen can influence the degradation rate [11] and, consequently, the amount of time it takes to change their properties [33].

The use of biodegradable polymers presents a promising opportunity for food packaging with regard to the initiative to protect the environment, as effective waste management not only involves efficient disposal but also the prevention of waste generation. In addition to their low environmental impact, fully biodegradable end-of-life packaging avoids recycling problems [39]. Furthermore, its biodegradable nature allows it to act as a soil conditioner and fertilizer [40].

There is a wide range of renewable carbon-based biopolymers that can be explored for their applicability in food packaging, but by far the most frequently studied and used are cellulose, starch, and chitosan, as well as soy proteins, gelatin, whey and casein [41]. This paper does not intend to expose these materials, as numerous previous studies have presented and synthesized information about them [4,16,30,42,43].

Although the potential of biodegradable materials is enormous, offering opportunities for resource protection and reducing environmental pollution, they present certain limitations that can hinder their acceptability. One of the drawbacks is the need for advanced technologies, resulting in higher costs compared to conventional materials and making the process of producing biodegradable packaging at an industrial scale more challenging [42]. Another concern may be the poor performance of the final barrier of the packaging [17]. In this regard, compatible polymer blends can enhance the performance of the material [36].

#### 2.2.3. Active Packaging for Food Products

Alongside the research on biodegradable materials, another step in the efforts for sustainable development in the food industry involves the shift towards preventing or delaying the spoilage process of food products through active packaging [27]. Innovations found in the food industry involve the interaction between food products and the active substances within the internal environment of the packaging [44]. In addition to extending the shelf life [45], another key point is highlighted, namely the enhancement of the food product’s value [46].

Therefore, in terms of food packaging, the focus shifts from being a simple passive barrier [47] to more complex technologies that ensure the quality of the packaged products [48].

Biodegradable packaging films can be used as both active and intelligent packaging [49]. Active packaging, as the name suggests, is the component that takes on certain actions or functions [50]. Intelligent packaging, on the other hand, integrates various systems that detect information regarding the products contained within the packaging; this information is provided in various forms (via sensors, pH indicators, or other data carriers) [29,50].

Moreover, packaging can be designed to be intelligent, signalling changes that occur in the condition of the packaged foods and indicating alterations in their quality or freshness [29,51]. The subject can also extend to the formulation of options that are organoleptically acceptable and thus safe for consumption if the active compounds are classified and registered as GRAS (generally recognized as safe) by the European Commission and the United States [52].

According to European Commission Regulation No. 450/2009, active systems “are intended to extend the shelf life or maintain or improve the condition of packaged food products; they are designed to deliberately incorporate components that would release substances into the packaged food products or into the environment in which the food products are located, or that would absorb substances from the packaged food products or from the environment in which the food products are located” [45]. Active packaging involves either the release of capture compounds (carbon dioxide, antioxidant agents, antimicrobial agents, enzymes, aromatic compounds, nutraceuticals) or the capture of emission compounds (oxygen, carbon dioxide, moisture, ethylene, odour) within the packaging content to inhibit degradation processes [1,24,27,45,48]. These substances provide antioxidant or antimicrobial activity when integrated into biopolymer films, which are particularly sensitive to moisture conditions [35].

Active packaging comes as a response to the growing demand for limiting processed products with preservatives [53]. Biopolymers offer a huge advantage, namely the ability to carry functional active substances [32]. Substances with antioxidant and antimicrobial properties include nanoparticles, essential oils or plant extracts, natural inorganic particles (oxides, nano-clays, metals), and phytonutrients [30,54]. Additionally, the active ingredients added to biodegradable films can contribute to enhancing their functionality [20]. Basically, the protection of packaged products is favoured by the presence of antioxidants and antimicrobial and antifungal substances [55].

To potentiate the desired effects, one can use variants of bioactive polymers which integrate antimicrobial characteristics, such as chitosan [26,51,56]. These properties are conferred by the active amino groups in its composition [57,58]. Other properties, such as solubility, viscosity, along with the ability to bind ions and form films, make it an ideal material for food packaging [59]. Its antimicrobial action mechanism involves interaction with free radicals, neutralizing them, and chelation of metal ions, binding and forming stable complexes that inhibit the catalytic activity of metals and reduce the formation of free radicals [60]. Other polymers with similar properties include carboxymethylcellulose, ε-polylysine, and poly ε-caprolactone [61].

Beyond the multitude of benefits provided by active packaging, it stands out as a necessity in the context of the development of trade and the transportation of goods, including food products. Microbiological contamination of food products is a major food safety issue that can lead to significant health risks for consumers, such as gastrointestinal, neurological, and immunological problems [43]. Products may contain various microbiological contaminants, which are estimated to affect 6 million cases and cause 420,000 deaths annually [29,38,43,62].

Preservatives and additives, although they offer significant advantages through low cost and demonstrated efficiency, raise questions regarding toxicity [63]. Concerns about their long-term use, along with the development of resistance among micro-organisms to synthetic antimicrobial substances, represent a significant concern in the field of food safety and public health [8]. In addition, some preservatives affect the sensory properties of foods, which is an undesirable consequence [64]. The focus is shifting from their use and traditional preservation techniques to establishing interactions between the packaging and the contained food product, contributing to sustainable and innovative solutions [63]. Thus, the integration of elements with antimicrobial and antioxidant potential becomes essential in protecting food, helping to maintain its quality and safety [10,64]. The field of these antibacterial substances is an extremely comprehensive one: natural antibacterial agents of plant origin, natural antibacterial agents of animal origin and natural antibacterial agents derived from micro-organisms [64].

Among the common means of obtaining packaging with antioxidant and antimicrobial potential are the use of polymers with such characteristics, the introduction of bioactive compounds into the packaging matrix, or their addition to the surface of the packaging [65]. At the same time, these compounds can be added in a layered manner within a permeable material and positioned close to the packaged food [66]. However, these impregnation methods, without coming into direct contact with the food’s surface, have the advantage of not imparting unwanted flavours or odours, and thereby not altering the organoleptic properties [67]. Interactions with the active agent in the matrix can be designed for controlled release so that it remains available for a longer time, extending the shelf life [68]. This extension leads to the postponement or delay of the expiration date, impacting both the consumer by preserving the nutritional value of purchased food products and the environment by reducing waste [39].

Along with efficient biodegradability to support the environment and maintaining the food quality through the integration of bioactive compounds, it is ideal for food films to reduce losses [69]. The alarming increases in annual food losses are a real cause for concern. Despite various existing preservation methods, global losses account for approximately 25% of the total food produced [68]. The highest waste occurs among fruits and vegetables (40–50%), followed by fish (35%) and cereals (30%), along with dairy products and meat (20%), with the main reason being represented by the processes of microbiological degradation [5]. Food spoilage involves affecting texture, loss of colour, and influencing nutritional value—aspects that contribute to reducing the quality of packaged products [25]. The shelf life of food basically refers to the period of time for which a food product maintains acceptable taste, colour, aroma, texture, nutritional value and safety when stored under specified environmental conditions [70]. The main purpose of active packaging focuses on three primary aspects: extending shelf life, maintaining quality, and ensuring safety [70].

Recently, the focus has shifted away from using antioxidant and antimicrobial substances on the surface of food products, as this can lead to their inactivation or evaporation [71,72]. The gradual release of antimicrobial agents provides protection and can enhance food safety by reducing the need for synthetic additives [73]. Controlled release ensures not only safety, but also an extended shelf life [70].

#### 2.2.4. Food-Contact Safety and Regulatory Considerations

According to Regulation (EC) No. 1935/2004 [74], food-contact materials must not transfer their constituents to food in quantities that could endanger human health, bring about unacceptable changes in food composition, or deteriorate the organoleptic characteristics of food. These requirements are particularly relevant for active food films, where active compounds are intentionally incorporated into the packaging material and may migrate into the food matrix. Therefore, the safety evaluation should not only assess the antimicrobial or antioxidant effectiveness of the film but also consider the identity and concentration of the active agent, its release rate, overall and specific migration, expected consumer exposure, and the conditions of use, including food type, storage time, temperature, and contact area. In real food applications, the migration and release of active compounds may depend on the food matrix, pH, fat content, moisture level, storage temperature, contact time, and surface-to-volume ratio.

Active and intelligent packaging materials are further regulated in the European Union by Regulation (EC) No. 450/2009 [75], which establishes specific requirements for materials designed to interact with food or provide information about food quality and safety [26]. Within this framework, active packaging components need to be safe for their intended use and must not mislead consumers, conceal food spoilage, or bring about unacceptable changes in food composition or sensory quality. These factors are especially significant for biodegradable films that include essential oils, plant extracts, phenolic compounds, or other volatile bioactive agents, since such substances can offer antioxidant or antimicrobial activity while also influencing the taste, odour, colour, or overall acceptability of packaged foods.

The regulatory status of active agents should also be considered.

In the United States, some essential oils, oleoresins, and natural extractives may be classified as generally recognized as safe (GRAS) for specific intended food uses. However, GRAS status should not be interpreted as an indicator of automatic approval for all active food-contact applications. When such compounds are incorporated into active food-contact films, their suitability still depends on the intended use, incorporated concentration, release behaviour, migration level, food matrix, storage conditions, and estimated dietary exposure.

Overall, biodegradable active food films intended for real food applications must be assessed through an integrated safety approach that combines functional performance with migration testing, toxicological evaluation, exposure assessment, sensory analysis, and compliance with relevant food-contact legislation. Although many natural active agents show promising antimicrobial and antioxidant properties, their practical use may be limited by excessive migration, strong sensory impact, instability during processing or storage, interactions with specific food matrices, or insufficient regulatory clearance. Addressing these safety and regulatory limitations is essential before such films can be transferred from laboratory-scale studies to commercial food-packaging applications.

#### 2.2.5. The Mechanism of Action of Antioxidant Substances

Understanding the main mechanisms of food degradation is essential for the development of biodegradable active food films, as these materials are designed not only to act as physical barriers, but also to incorporate active compounds capable of delaying microbial growth, oxidation, and other quality deterioration processes.

The food degradation process is influenced by both intrinsic factors (micro-organisms, pH, water activity, level of reactive compounds and enzymes) and extrinsic factors (temperature, total pressure, relative humidity, light, partial pressure of various gases and mechanical stress) [76].

The degradation process of food products also includes oxidation without exception. This occurs because oxygen is highly reactive and can interact with various compounds in food, leading to changes in quality, safety, and nutritional value [44]. Oxidation particularly affects lipids and proteins, contributing to their denaturation [43]. In addition to imparting undesirable organoleptic characteristics, compounds produced during these processes (such as ketones, aldehydes, and aromatic amines) may have toxic effects on the body [20,77]. Fat-rich products with a high degree of unsaturation are especially susceptible to these effects [78]. Lipid peroxidation causes both physical and chemical changes [77], leading to the loss of their functional and nutritional value [79]. The incorporation of antioxidants contributes to delaying the oxidation process in lipids, as well as the denaturation of proteins [77].

#### 2.2.6. The Mechanism of Action of Antimicrobial Substances

In addition to the concept of packaging with antioxidant properties, a significant proportion of studies address the antimicrobial qualities they present [5,8,25,26,38,46,56,63,64,80,81,82,83,84].

The main objective of active packaging is to enhance food safety by limiting microbial contamination and spoilage on food surfaces [25,85]. Another promising property of natural antimicrobial compounds is their ability to induce changes in the cell membrane of micro-organisms and attack the cell wall, leading to cell lysis [15,20,86]. Antimicrobial films contribute to extending shelf life by inhibiting the growth of micro-organisms [60].

The growing use of biopolymer-based films highlights their strong potential as a suitable platform for the implementation of natural active agents in sustainable food-packaging systems [25]. Incorporating antimicrobial agents into polymeric films enhances their hydrophobic properties, which leads to an increased transfer coefficient and a reduction in water vapour permeability (WVP) [70]. Such systems can take various forms [45]: sachet-type packages for releasing volatile substances, films, or edible coatings that cover the food products [70]. The latter are thinner and maintain continuous contact with the packaged product, falling under both the category of packaging and food products [87].

To achieve antioxidant and antimicrobial properties, packaging typically contains three classes of chemical compounds: biopolymers to structure the base matrix (polysaccharides, proteins or lipids) and preservation components, which can be of natural or synthetic origin and even additives (emulsifiers, plasticizers) [88]. Therefore, the combination of biodegradable polymers and active technologies has excellent prospects for exploration in the coming years, contributing to the improvement of packaging systems in the food industry [89].

This can be achieved either by directly incorporating antioxidant or antimicrobial substances (extracts, essential oils, nanocomposites) into the packaging film matrix applying them to the surface, or using biodegradable polymers with such properties [10]. At the same time, active substances can be encapsulated in the matrix using colloidal particles [81]. Encapsulation is also applicable to anthocyanins, which can be used as bioactive compounds in packaging, keeping their properties intact [90].

**Figure 7:**
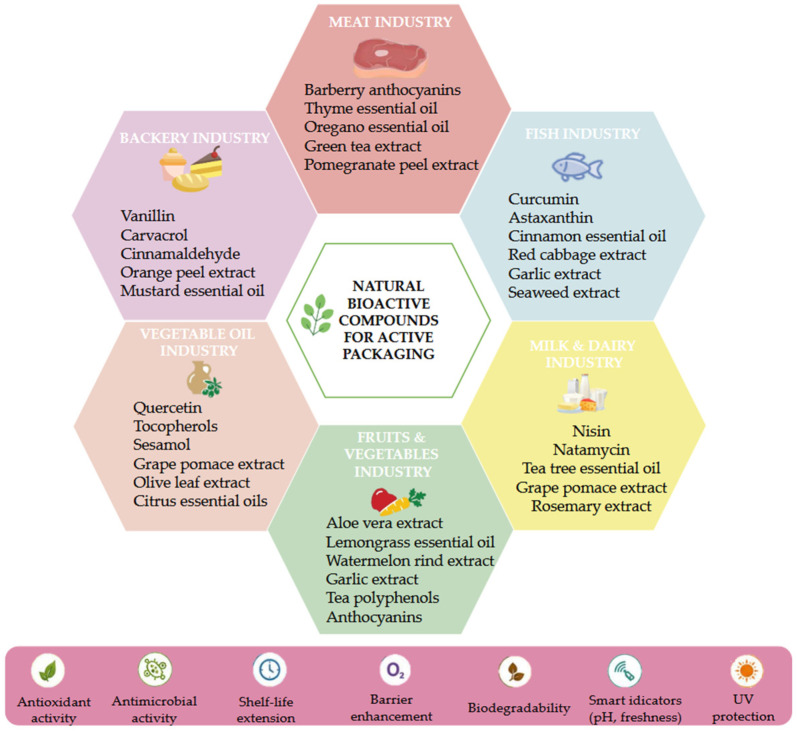
Representative natural bioactive compounds used in active food-packaging applications across different food industries.

Many studies focus on the incorporation of natural extracts [9,20,21,46,69,91,92], essential oils [14,41,93,94], nanocomposites [17,31,43,57,95,96] or even by-products [32,51,86,97,98] into biodegradable packaging, highlighting their benefits and applicability in the food industry (Figure 7).

Nanoparticles are also used as part of active or intelligent packaging, taking various forms such as nanotubes, nanoemulsions, nanocomposites, nanocapsules, and nanofibers [99]. These nanomaterials serve functions as antimicrobial agents, oxygen scavengers or provide information about storage conditions in active and intelligent packaging [5]. They involve the integration of metal ions or metal oxides (silver, copper oxide, zinc oxide, and titanium dioxide), which also confer antimicrobial properties to the final packaging [7]. A generally accepted mechanism for the antimicrobial activity of metal oxides is based on differences in electrical charge: micro-organisms carry a negative charge, while metal oxides have a positive charge, leading to an attraction that results in the oxidation and destruction of micro-organisms [100]. Composite films containing metallic particles or carbon-based materials exhibit active properties by capturing undesirable substances, providing both antimicrobial and antioxidant effects [101].

Essential oils offer significant advantages that make them suitable for use in packaging, such as being able to be introduced in the form of nanoparticles or microcapsules, which increases their stability, solubility, and effectiveness [102]. Encapsulation enhances their physicochemical properties, making the oils less sensitive to factors such as light, oxygen, and temperature changes [11,103]. Studies indicate that it is possible to use multiple active compounds to achieve better synergy, e.g., by combining essential oils or constituents of essential oils [89]. Essential oils penetrate bacterial cell walls and cytoplasmic membranes, destabilizing the membrane and fluidizing it, which can lead to the leakage of essential ions and molecules, ultimately resulting in cell death [104].

Recent studies indicate that there is potential for integrating probiotics or prebiotics into active films through technologies that protect their viability and functionality during storage [87]. In general, biopolymers based on polysaccharides and proteins are very commonly used in research and development due to their biocompatibility, biodegradability and versatility [43].

To explore the possibility of producing packaging from natural sources and to reduce the environmental impact of the process, the valorization of by-products and waste from the agri-food industry is being considered [39]. In this way, by-products take on the role of functional ingredients, increasing the value of the films when used in their composition [30]. Their constituents, which are rich in polysaccharides, proteins and/or lipids, make them ideal as a basis for food film matrices [30]. Additionally, this wide range can provide polyphenols and other bioactive compounds that support the active potential of biodegradable packaging [11], alongside generating improved physical, mechanical and barrier properties [32,89].

Increasing the availability of natural ingredients can be achieved by utilizing conventional sources of industrial waste, such as oil seed cakes or sugarcane bagasse, as well as non-conventional sources, including extracts from oilseed peels, rhizomes, and grains [105]. One example is grape seed extract, which, when introduced into biodegradable films, significantly reduces the growth rate of micro-organisms and the rate of fat oxidation [21]. Also, watermelon rind has the potential to provide antioxidant and antimicrobial properties against food pathogens in freshly cut red cabbage [69]. Incorporating lemon peel into PVA and starch films contributed to a significant improvement in their thermal stability and antioxidant activity [106]. The use of by-products derived from leaves, seeds, peels or unused pulp in food packaging also contributes to shaping a more sustainable food industry [103]. In this broad context of by-products, their value increases considerably due to their high frequency of availability, making them a valuable source of bioactive compounds such as vitamins, minerals, fibres and phenolic compounds [86].

By-products integrated into the matrix of food films not only serve as an affordable and advantageous alternative due to their inherent properties but also support the circular economy. These materials are no longer regarded as waste but rather as resources with high valorization potential, aligning well with the “zero waste” concept [97]. Furthermore, they contain valuable biopolymers such as polysaccharides and proteins, as well as bioactive compounds like flavones, flavonoids, phenols, phenolic acids, coumarins, terpenoids, alkaloids, quinones, tannins, lectins and phytosterols [9,15]. On the other hand, inadequate management of such waste may lead to greenhouse gas emissions and the buildup of micro-organisms, including pathogens [98].

To minimize contamination and extend the shelf life of foods, numerous studies have been conducted to develop packaging options adaptable to the specific requirements of food products. Examples of this are shown in Table 4, which presents a selection of bioactive agents recently integrated into food films, along with the results obtained.

Table 4 shows a comparative summary of recent studies on biodegradable active films, covering the polymer matrix, active agent, target food or application, tested function, storage or shelf-life outcome, biodegradation assessment, and main limitations. Overall, the studies indicate a clear move from simple biodegradable films to multifunctional active packaging systems. Chitosan, starch-based materials, gelatin, alginate, cellulose derivatives, PVA, PCL, PBAT, and PLA are among the most frequently studied matrices, while plant extracts, polyphenols, essential oils, natural pigments, and nanoparticles are commonly used as active agents [8,69,83,92,122,123]. Beyond biodegradability, recent research has examined antimicrobial and antioxidant activity, improved barrier and mechanical properties, controlled or gradual release of active compounds, and shelf-life extension in specific food products.

A closer comparison of the studies in Table 4 shows that natural polymer matrices and biodegradable synthetic polymers each have their own advantages and drawbacks. Chitosan, starch, gelatin, alginate, cellulose derivatives, and seed gums are often used because they are biodegradable and can easily carry natural active compounds. Still, these films often need better water resistance, mechanical strength, or barrier properties. In contrast, PLA, PCL, PBAT, and related blends usually provide stronger mechanical or barrier performance, but they often require added active compounds or nanoparticles to achieve strong antioxidant or antimicrobial effects [22,72,118,120,123].

The active compounds also function in different ways. Plant extracts, polyphenols, anthocyanins, and green tea extract mainly improve antioxidant activity by reducing free radicals, binding metal ions, or slowing lipid oxidation. Essential oils and some plant compounds mainly inhibit micro-organisms by damaging microbial membranes or limiting microbial growth. Nanoparticles such as ZnONPs, AgNPs, and Ag-MMT can improve antimicrobial activity through ion release or direct interaction with microbial cells, but they also need careful safety evaluation because migration into food may occur [17,22,120].

Another notable difference is the level of practical validation. Some studies examined the release of active compounds in water or food simulants, such as green tea extract from PCL/PLA films or Echinacea extract from AHSG films, while others also considered migration-related aspects, such as zinc release from chitosan/ZnO films [22,72,123]. Several studies applied biodegradable active films to real food systems, such as meat and poultry products [8,22,122,123], fish or seafood [80,119,120], dairy products [17], sausages [72], and fresh-cut vegetables [69,92]. However, the storage time, spoilage indicators and sensory evaluation varied greatly among these studies. In addition, many papers described the films as biodegradable because of the polymer used, but only a few included an experimental biodegradation test. Therefore, future studies should include more realistic storage tests, standardized biodegradation conditions, release or migration studies in real foods, and sensory analysis before these films can be used commercially.

## 3. Discussion

The marked growth in the number of publications during the analyzed period, which nearly doubled between 2021 and 2025, indicates the increasing relevance of active biodegradable food films within food-packaging research. This trend reflects the growing scientific interest in developing sustainable packaging systems capable of combining environmental benefits with antimicrobial and antioxidant functionalities.

The keyword co-occurrence analysis shows that studies on active biodegradable food films are centred on several connected directions, including functional film properties, biopolymer-based matrices, bioactive compounds, edible films, and advanced material systems. This organization reflects the multidisciplinary nature of the field and suggests a move toward multifunctional packaging systems that combine biodegradability with antimicrobial and antioxidant protection. Future studies should also pay greater attention to the stability of active compounds, their controlled release behaviour, material performance, and validation under real food storage conditions.

The thematic map shows that research on active biodegradable food packaging is now centred on topics like biodegradable films, food packaging, active films, and essential oils, emphasizing how sustainability and food preservation functions come together. At the same time, antibacterial and antioxidant activities remain key research fields that support the progress of active packaging systems. The appearance of less-developed themes such as anthocyanin-based indicators and smart packaging points to chances for more innovation, especially in creating multifunctional packaging materials that can both protect food and track its quality.

The distribution of publications across journals in materials science, polymer science, food technology, food chemistry, and packaging research shows the interdisciplinary nature of active biodegradable food films. The strong presence of journals like International Journal of Biological Macromolecules and Polymers highlights the rising importance of biopolymeric and sustainable materials research, while Food Packaging and Shelf Life and Food Chemistry stress the significance of applications connected to food preservation and packaging performance. In addition, the broad spread of publications across various scientific areas supports knowledge exchange and may encourage future collaborations focused on creating more effective and sustainable packaging solutions.

During the selected 2021–2025 period, the authorship analysis shows that research on active biodegradable food films is quite collaborative, as demonstrated by the average number of co-authors per paper and the wide spread of contributions among many researchers. The observation that even the most productive authors produced only a modest number of papers suggests the field is not controlled by a small circle of researchers, but rather shared across a broader international research community. This collaborative pattern likely supports the combination of knowledge from materials science, food technology, chemistry, and packaging research, which is important for developing multifunctional biodegradable active films.

The geographical distribution of publications shows that research on active biodegradable food films is mainly driven by countries with strong activity in the fields of food science, biomaterials, and sustainable technologies. The leading role of China, followed by Brazil and India, points to a growing global interest in developing environmentally friendly packaging systems. The involvement of countries from various regions also emphasizes the international nature of this field and may support the exchange of knowledge, technologies, and research methods aimed at tackling sustainability and food preservation challenges.

Overall, the bibliometric findings indicate that future research on biodegradable active food films should move on from focusing just on material development to more integrated studies that combine bio-based polymer matrices, natural active compounds, controlled release behaviour, real-food validation, safety assessment, and sustainability evaluation. The observed publication patterns, keyword clusters, thematic shifts, and collaboration networks show that the field is progressing toward multifunctional packaging systems meant to preserve food quality and lessen environmental impact. Therefore, future studies should stress scalable film-forming technologies, long-term storage performance, biodegradation under realistic conditions, and closer links between laboratory results and real food-packaging practices. While the bibliometric analysis provides insights into publication trends, collaboration patterns, and thematic developments within the field, the narrative review offers a more detailed perspective on the materials, active compounds, and technological approaches currently being investigated for the development of biodegradable active food films.

Although the reviewed studies show promising results, several challenges still exist. Many formulations have been tested mainly in laboratory settings, and data on long-term stability, large-scale production, economic viability, and performance under actual food storage conditions remain scarce. These factors mark key directions for future research and industrial application.

Therefore, the broad scope covered by active biodegradable packaging in recent research cannot be overlooked. The findings highlight the importance, diversity and even the necessity of integrating these solutions into the food sector as ongoing research explores new possibilities for developing films with enhanced structural and compositional properties. In this context, active biodegradable food films may contribute to both food-quality preservation and environmental sustainability.

This study has certain limitations that should be recognized. The analysis relies solely on publications obtained from the Scopus database and covers only the 2021–2025 period. In addition, the findings represent bibliometric indicators and publication patterns rather than the methodological quality of each study. Thus, the results should be viewed as a general overview of the research field and the thematic development of active biodegradable food films.

## 4. Materials and Methods

The increase in scientific research over recent decades has been accompanied by a growing interest in bibliometric analysis. This approach has attracted more attention lately, as it allows the visualization of scientific productivity on a global scale and the evaluation of research quality. Using mathematical and statistical methods, it becomes possible to identify trends within the chosen research area. The bibliometric analysis presented in this study points out key research trends and showcases the current state of knowledge about active and biodegradable packaging in the food industry.

**Figure 8:**
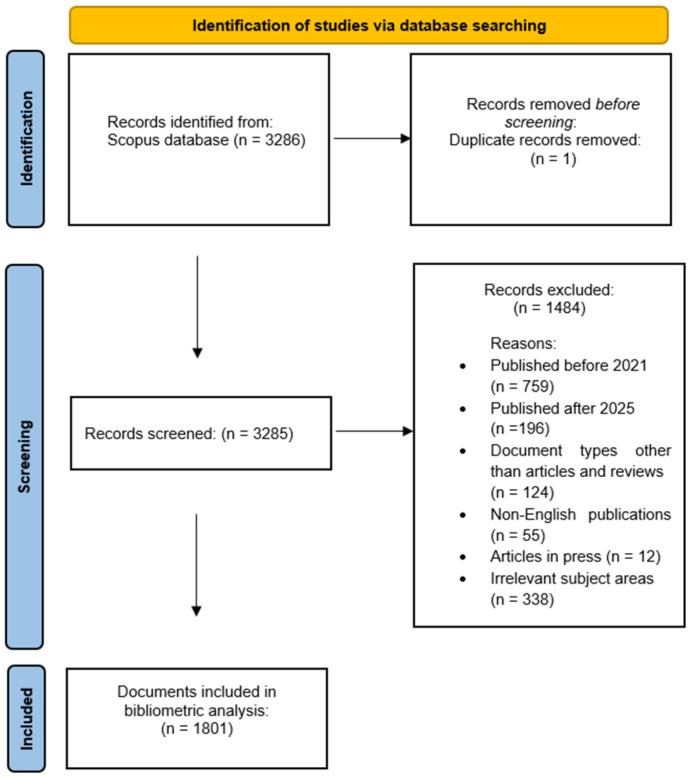
PRISMA-based flow diagram illustrating the identification and filtering process applied to the Scopus dataset [124].

To ensure transparency and systematize the study selection process, a flow diagram was developed according to the PRISMA methodology, as shown in Figure 8.

The flow diagram shows the Scopus search and filtering process used to obtain the final dataset of 1801 records for the bibliometric analysis. It serves as a PRISMA-based summary of the selection process, not as a full systematic review protocol.

The relevance of the dataset was mainly ensured through the search strategy and the Scopus filters applied, which included the publication period, document type, language, and subject area. These criteria helped to keep publications focused on sustainable and active food packaging. The final dataset, containing 1801 records, was then used for the bibliometric analysis. For the narrative literature review, relevant and representative studies from this dataset were manually reviewed, and particular attention was paid to biodegradable active food films, active compounds, food-packaging applications, and preservation performance.

The selection of studies for detailed discussion centred on publications describing clear film-forming matrices, active compounds, preparation methods, target food products or micro-organisms, and functional properties such as antimicrobial, antioxidant, mechanical, barrier, or preservation performance. Studies lacking sufficient methodological details or those not directly related to biodegradable active food films were excluded from the detailed discussion. The same criteria guided the choice of representative examples presented in Table 4.

### 4.1. Data Collection and Selection Criteria

The present study is based on the specialized literature retrieved from the Scopus database in order to understand the current state of research on the development of biodegradable films with active properties applicable in the food-packaging industry. In addition, the literature analysis can help to identify research gaps, there by contributing to future studies [19].

Numerous studies have addressed the topic of packaging in the food industry; however, not all of them include aspects related to active properties [24]. During the data collection stage, careful selection of keywords found in titles and abstracts is essential, as it represents an important inclusion criterion. The search results must be relevant, focused, and sufficiently comprehensive to cover all relevant aspects of the topic.

The bibliometric dataset was retrieved from the Scopus database on 12 May 2026. The last five years (2021–2025) were selected due to the rapid development of sustainable and active technologies.

The search was conducted in the Scopus database using the “Title–Abstract–Keywords” field. The search string was structured as follows: (“active film” OR “biodegradable film” OR “bio-based film”) AND (“food” OR “preservation” OR “shelf life”). The search was restricted to the following Scopus subject areas: agricultural and biological sciences, chemistry, biochemistry, genetics and molecular biology, materials science, engineering, chemical engineering, and environmental science.

In the first stage, 3286 records were retrieved from the Scopus database. In the second stage, a screening process was applied to refine the dataset and ensure its relevance and topical focus. The dataset was restricted to publications from the last five years (2021–2025) to ensure coverage of the most recent developments in sustainable and active packaging technologies. Duplicates and non-English publications were excluded from the initial dataset. In addition, only articles and review papers were included, and conference papers, book chapters, and other document types were excluded to ensure that only peer-reviewed and finalized studies were considered. The final dataset of 1801 records was used for the bibliometric analysis, while the literature review focused on selected relevant and representative studies related to biodegradable active food films, active compounds, food-packaging applications, and preservation performance.

### 4.2. Content Analysis

The data obtained from the Scopus database were exported in CSV format, compatible with R version (4.3.3 (2024-02-29 ucrt)) and analyzed using the Biblioshiny graphical interface of the Bibliometrix R package implemented in RStudio (Version 2026.04.0+526). The same dataset (CSV format) was also used in VOSviewer (software version 1.6.20) for constructing and visualizing term networks and citation maps in scientific research [24]. These two bibliometric tools enable the analysis of selected publications and the evaluation of research impact in the field, which may further contribute to the identification of potential research gaps. Both programmes rely on cartographic visualization techniques, which are essential for exploring the intellectual structure of a specific research domain, namely biodegradable active packaging. This study analyzed annual scientific production, publication distribution patterns, keyword co-occurrence networks and thematic clusters, as well as the most relevant authors, countries, and scientific contributions in the field. These analyses were used to identify research trends, intellectual structures, and emerging directions in biodegradable active food-packaging research.

## 5. Conclusions

This study combined a narrative review with a bibliometric analysis to present an updated overview of biodegradable active food films published between 2021 and 2025. The bibliometric results revealed a steady rise in publication numbers, confirming the growing interest in sustainable food packaging with antimicrobial and antioxidant properties. The analysis also highlighted the main research themes in this area, such as biodegradable films, active packaging, bioactive compounds, and food preservation. Countries like China, Brazil, and India made significant contributions to this research field.

Taken together, the bibliometric findings and the critical review suggest that future research on biodegradable active food films should go beyond material development alone. More integrated studies are required, combining bio-based polymer matrices, natural active compounds, controlled release behaviour, real-food validation, safety testing, and sustainability assessment. The publication patterns, keyword groupings, thematic shifts, and collaboration networks indicate that the field is moving toward multifunctional packaging systems aimed at maintaining food quality and reducing environmental impact.

Although notable progress has been achieved, several gaps still exist. Many studies remain at the laboratory scale, while industrial production and large-scale applications are less explored. The controlled release of active compounds also deserves more attention, as it is essential for maintaining film activity during storage and preventing excessive migration or changes in food quality. Furthermore, many studies assess storage performance only for short durations or on a single food product.

Future research should concentrate more on practical testing under real conditions. Biodegradable active films need to be evaluated on actual foods over longer storage periods, including microbial, oxidative, and sensory assessments. Migration or release studies are also necessary to confirm food-contact safety. In addition, biodegradation should be tested under realistic environments, such as soil or composting, rather than assuming biodegradability solely from the polymer used. Overall, biodegradable active films hold strong potential for food preservation and sustainable packaging, but their future application depends on better validation of performance, safety, scalability, and degradation behaviour after disposal.

## Figures and Tables

**Table 2:** Top 10 most productive authors in research on biodegradable active food films.

No.	Authors	Articles
1	Xie, Jing	20
2	Harnkarnsujarit, Nathdanai	18
3	Chougale, Ravindra B.	16
4	Masti, Saraswati P.	16
5	Jamróz, Ewelina	15
6	Chen, Chenwei	14
7	Chiralt, Amparo	14
8	Zhang, Wanli	14
9	Almasi, Hadi	13
10	Marangoni Júnior, Luís	13

**Table 3:** Top 10 contributing countries in research on biodegradable active food films.

No.	Region	Number of Contributions
1	China	3031
2	Brazil	1313
3	India	979
4	Iran	648
5	Spain	327
6	Italy	296
7	Poland	267
8	Portugal	266
9	Thailand	253
10	Malaysia	228

**Table 4:** Food films developed with active compounds incorporated into the packaging matrix.

Polymer Matrix	Active Agent	Target Application	Tested Function	Main Outcome	Biodegradation	Main Limitation	References
Carrageenan	Jabuticaba (grape tree) peel extract	General active/intelligent food-packaging application; suggested for fish freshness monitoring	Antioxidant activity; antimicrobial activity; pH-sensitive colour indicator; UV–vis light barrier; water vapour barrier	Antioxidant activity up to 58.91%; microbial inhibition against *E. coli*; visible colour changes at different pH values; reduced WVP and swelling index; potential use as freshness indicator	Not experimentally evaluated	No real-food validation; fish application suggested only for future work; migration/release and sensory impact not assessed	[91]
Pea starch and cellulose nanofibers	Apple polyphenols	General active food-packaging application; UV-exposure-sensitive foods suggested	Antioxidant activity; UV-blocking ability; mechanical properties; thermal stability; biodegradability	DPPH radical-scavenging activity reached 73.77%; improved UV-blocking ability and thermal stability; biodegradation ratio increased up to 45.30% after 20 days	Soil burial test; mass loss after 3, 10 and 20 days	No real-food validation; no storage/shelf-life test on food; migration/release and sensory impact not assessed	[18]
PVA	Piperic acid derived from black pepper	General active food-packaging application	Antibacterial activity; antifungal activity; oxygen and water vapour barrier; mechanical properties; thermal stability; hydrophobicity	Antibacterial activity against *S. aureus* and *E. coli*; antifungal activity against *Fusarium solani*, *Penicillium* and *Aspergillus*; improved OTR, WVTR, tensile strength, thermal stability and water resistance	Not experimentally evaluated	No real-food validation; no storage/shelf-life test on food; migration/release and sensory impact not assessed	[107]
Chitosan	Blueberry, red grape and parsley by-product extracts	General edible/active food-packaging application	Antioxidant activity; antimicrobial activity; oxygen barrier; migration of bioactive compounds; physical and textural properties	Highest antimicrobial activity against *E. coli* for red grape extract films; improved oxygen barrier; increased antioxidant properties; reduced swelling degree with higher extract concentration	Biodegradability mentioned from previous related work, but not experimentally assessed in this study	No real-food validation; no storage/shelf-life test on food; sensory analysis not included in this study	[52]
PVA	Hydroxytyrosol and oleuropein extracts from olive oil pomace	General active food-packaging application; oxidative-sensitive foods	Antioxidant activity; UV-light barrier; specific migration/release in food simulant; optical and mechanical properties	Strong antioxidant activity in films and food simulant; reduced UV transmittance; PVA/OleE-10 showed the highest antioxidant activity; rapid release of active compounds during the early contact period	Not experimentally evaluated	No real-food validation; no storage/shelf-life test on food; biodegradation and sensory impact not assessed; release was assessed in food simulant rather than in a real food matrix	[97]
Gelatine	Green tea extract and lemon essential oil nanoemulsion	General edible/active food-packaging application	Water vapour permeability; water solubility; mechanical properties; thermal stability; FTIR interactions	Green tea extract increased tensile strength; lemon nanoemulsion and green tea extract modified WVP, solubility and thermal behaviour; potential use as edible film	Not experimentally evaluated	No real-food validation; no storage/shelf-life test on food; antioxidant/antimicrobial activity, migration/release and sensory impact not directly assessed in this study	[94]
PVA and corn starch	Lemon peel powder	general active food-packaging application; oxidation-sensitive foods	antioxidant activity; thermal stability; film thickness; surface morphology	lemon peel improved antioxidant activity and thermal stability; films with 8% lemon peel showed the highest radical-scavenging capacity; potential use for oxidation-sensitive food preservation	not experimentally evaluated	no real-food validation; no storage/shelf-life test on food; migration/release, sensory impact and antimicrobial activity not assessed	[106]
Orange peel-based film	Arabic gum and chromium oxide nanoparticles	General active food-packaging application	Antimicrobial activity; water vapour permeability; mechanical properties; thermal stability; morphology; FTIR and XRD analysis	Gum Arabic and Cr_2_O_3_ nanoparticles improved antimicrobial activity and mechanical properties; Cr_2_O_3_ increased thermal stability; inhibition zones were observed against *S. aureus* and *E. coli*	Not experimentally evaluated	No real-food validation; no storage/shelf-life test on food; migration/release, sensory impact and nanoparticle safety in food-contact conditions not assessed	[6]
Alginate	Date palm pit extract (DPPE)	General active food-packaging application; foods rich in unsaturated fats, such as nuts and seeds, suggested	Antioxidant activity and stability; water vapour barrier; solubility; surface wettability; mechanical and optical properties	DPPE improved antioxidant and light-barrier properties; 10–20% DPPE improved WVP and mechanical strength; 40% DPPE showed the lowest loss of phenolic content and antioxidant activity after 90 days	Not experimentally evaluated	No real-food validation; no storage/shelf-life test on food; migration/release and sensory impact not assessed	[108]
Cellulose acetate and PCL	Copper nanoparticles	General active food-packaging application; postharvest food protection suggested	Antimicrobial activity; hydrophobicity/contact angle; water vapour and gas permeability; mechanical properties; cytotoxicity	Highest CuNP-loaded film (F-Cu-3) showed strongest antimicrobial activity, 160° contact angle and reduced WVTR	Not experimentally evaluated	No real-food validation; no storage/shelf-life test on food; migration/release of CuNPs and sensory impact not assessed; safety evaluation limited to in vitro cytotoxicity	[109]
Sodium caseinate, xanthan gum and konjac glucomannan	Clove essential oil (CEO)	General active food-packaging application; fresh fruits, vegetables and meat products suggested	Optical/UV-barrier properties; hydrophobicity/contact angle; mechanical properties; rheological behaviour; morphology; FTIR, XRD and thermal stability	CEO improved UV-blocking ability and surface hydrophobicity; contact angle increased up to 78° at 0.8% CEO; films showed dense structure and potential antioxidant/antibacterial functionality	Not experimentally evaluated	No real-food validation; no storage/shelf-life test on food; antioxidant/antimicrobial activity, migration/release, sensory impact and biodegradation not directly assessed	[14]
Modified gluten	*Heracleum persicum* essential oil, magnesium oxide nanoparticles and polypyrrole	General active/intelligent food-packaging application; perishable foods suggested	Antioxidant activity; electrical conductivity; solubility; moisture content/absorption; WVP; mechanical, structural and thermal properties	Film showed the highest antioxidant activity; polypyrrole increased electrical conductivity and thermal resistance; additives improved film structure and potential active/intelligent packaging performance	Not experimentally evaluated	No real-food validation; no storage/shelf-life test on food; antimicrobial activity, migration/release, sensory impact and biodegradation not directly assessed	[71]
Cellulose acetate	*Falcaria vulgaris* extract	General active food-packaging application; shelf-life extension suggested	Antioxidant activity; antimicrobial activity; water vapour and oxygen barrier; contact angle; water absorption/swelling; mechanical properties; opacity/colour; biodegradability	Films with 0.3% extract showed improved antioxidant activity, antimicrobial activity against *E. coli*, *S. aureus*, *C. albicans* and *C. glabrata*, a higher contact angle, reduced WVP/water absorption and high biodegradation after 75 days	Oil burial test; 65–95% weight loss after 75 days, depending on *Falcaria vulgaris* extract concentration	No real-food validation; no storage/shelf-life test on food; migration/release and sensory impact not assessed; mechanical strength decreased with extract addition	[110]
Dextrin and PVA	Titanium dioxide nanoparticles	General bioactive food-packaging application; light-sensitive/lipid-based foods suggested	Antioxidant activity; antibacterial activity; biodegradability; water solubility; water vapour permeability; mechanical properties; transparency; contact angle; thermal stability	TiO_2_ improved tensile strength, contact angle, thermal stability and antioxidant activity; antioxidant activity reached about 41% in 95% ethanol; films showed selective antibacterial activity against *Enterococcus faecalis*	Soil burial test; C3 film showed the highest biodegradation, about 97% weight loss after 60 days	No real-food validation; no storage/shelf-life test on food; migration/release of TiO_2_/citric acid and sensory impact not assessed; antibacterial effect was selective, mainly against *E. faecalis*	[7]
Cassava starch (Tapioca)	Tucumã oil and tucumã oil microparticles obtained by spray drying, spray chilling and combined spray drying/spray chilling	General active food-packaging application; light- and oxidation-sensitive foods suggested	Optical/light-barrier properties; colour stability; water solubility; moisture content; mechanical properties; microstructure; carotenoid retention	Tucumã oil and microparticles increased yellow colour and opacity; enriched sheets showed reduced water solubility; FSDC sheets showed better colour stability after 60 days, suggesting improved carotenoid protection	Not experimentally evaluated	No real-food validation; no storage/shelf-life test on food; antioxidant activity, carotenoid migration/release and sensory impact not assessed; extrusion conditions and mechanical properties require optimization	[111]
Chitosan	Cashew nut shell liquid, iron oxide nanoparticles and plant extracts from *Mentha spicata*, *Vitex negundo* and *Eucalyptus globulus*	General active/barrier food-packaging application; oxygen-sensitive and lipid-containing foods suggested	Antibacterial activity; UV-barrier property; oxygen barrier; thermal stability; morphology/structural properties	Improved UV-blocking and oxygen-barrier properties; OTR decreased from 434.927 to 160.22 cc/m^2^·day; antibacterial resistance against *E. coli*, *P. aeruginosa*, *B. subtilis* and *S. aureus*	Not experimentally evaluated	No real-food validation; no storage/shelf-life test on food; migration/release, sensory impact, antioxidant activity and biodegradation not directly assessed	[112]
Potato starch	Thymol (Thy) encapsulated in mesoporous nano-silica	General active food-packaging application; aqueous, acidic and fatty food simulants tested	Antioxidant activity; sustained thymol release; UV-blocking property; water vapour permeability; moisture absorption; mechanical properties; thermal stability	0.5% Thy-MCM-41 optimized film properties, increasing TS to 4.01 MPa and reducing MA and WVP; films showed antioxidant activity and sustained thymol release, with the highest release in 50% ethanol simulant	Not experimentally evaluated	No real-food validation; no storage/shelf-life test on food; sensory impact and biodegradation not assessed; excessive Thy caused particle agglomeration and poorer film properties	[113]
PLA bilayer films containing cellulose nanocrystals or lignin nanoparticles	Umbelliferone combined with cellulose nanocrystals or lignin nanoparticles	General active food-packaging application; light- and oxidation-sensitive foods suggested	Antioxidant activity; UV-light barrier; optical properties; mechanical properties; thermal stability; morphology	Bilayer films showed good interfacial adhesion and improved stress at break; LNP and UMB reduced UV transmission; PLA_3LNP/PLA_15UMB showed the highest antioxidant activity among bilayers due to the synergistic effect of lignin nanoparticles and umbelliferone	Not experimentally evaluated	No real-food validation; no storage/shelf-life test on food; migration/release, sensory impact, antimicrobial activity and biodegradation not directly assessed	[114]
Nanocellulose and carboxymethylated nanocellulose	Thyme, cinnamon and oregano essential oils	General active food-packaging application; headspace/vapour-phase antimicrobial release suggested	Essential oil sorption and solubility; release kinetics; diffusivity; FTIR interaction analysis	Essential oil release could be tuned by the nanocellulose carboxymethylation degree; thyme showed the highest solubility, about 20 wt%; vapour release showed an initial fast stage followed by slower prolonged release	Not experimentally evaluated	No real-food validation; no storage/shelf-life test on food; antimicrobial activity, sensory impact and biodegradation not directly assessed; study focused on diffusion/release behaviour rather than food preservation performance	[115]
Fishskingelatin	Guava leaf extract (*Psidium guajava*) and catechin	General edible/active food-packaging application; broad food-contact use suggested	Antioxidant activity; antibacterial activity; UV-barrier property; mechanical properties; water vapour permeability; solubility; thermal stability; morphology	Catechin/guava leaf extract films showed strong antioxidant activity and antibacterial inhibition against *E. coli*, *S. aureus*, *B. cereus* and *S. typhimurium*; 30:70 catechin/guava leaf extract ratio showed the best overall performance	Not experimentally evaluated	No real-food validation; no storage/shelf-life test on food; migration/release and sensory impact not assessed; transparency, colour, thickness and WVP still require improvement compared with PVC	[116]
Sodium caseinate reinforced with bacterial cellulose and halloysite nanotubes	*Rosa damascena* essential oil (REO)	Strawberries; active edible food-packaging application	Antibacterial activity; strawberry preservation test; WVP; moisture absorption; contact angle; mechanical properties; thermal stability; morphology	REO improved antibacterial activity against *E. coli* and *S. aureus* and delayed strawberry spoilage during 7 days at room temperature; BC2H1/EO and BC2H3/EO were promising formulations	Not experimentally evaluated	Food validation was limited to visual strawberry storage; no quantitative shelf-life/microbial analysis on food, migration/release study, sensory evaluation or biodegradation test was reported	[104]
Chitosan	Ginseng residue polysaccharides (GRPs)	Fresh-cut melon; antioxidant active packaging	Antioxidant activity; fresh-cut melon preservation; weight loss; firmness; titratable acidity; soluble solids; malondialdehyde; ascorbic acid; total phenols; catalase activity; DPPH; WVP; mechanical, thermal and structural properties	Chitosan film with 0.5% GRP delayed deterioration of fresh-cut melon during 4 days at 25 °C and 55% RH; better retained quality and antioxidant status than plastic film and neat chitosan film	Not experimentally evaluated	Storage validation was limited to fresh-cut melon for 4 days; migration/release of GRP, sensory effects, microbial shelf-life analysis and biodegradation were not directly assessed	[117]
*Zanthoxylum bungeanum* leaf powder	Native polyphenols and flavonoids from *Zanthoxylum bungeanum* leaves (ZBL)	Fresh-cut apple; antioxidant active packaging	Antioxidant activity; fresh-cut apple preservation; HPH effect; WVP; oxygen permeability; water solubility; contact angle; mechanical, optical, thermal and morphological properties	Film with 1.5% leaf powder showed improved mechanical strength, lower WVP and denser microstructure; fresh-cut apples packaged with HPH-treated ZBL films maintained better quality during 5 days at 4 °C and 70% RH	Not experimentally evaluated	Storage validation was limited to fresh-cut apple for 5 days; migration/release, sensory evaluation, biodegradation and microbial shelf-life analysis were not directly assessed; HPH treatment may reduce part of the antioxidant capacity	[9]
Chitosan	Tulsi essential oil (TEO)	Fried potato fingers; antioxidant active packaging for lipid oxidation-sensitive foods	Antioxidant activity; oxidative stability of fried potato fingers; DPPH and H_2_O_2_ radical scavenging; FTIR oxidation analysis; WVTR; moisture content; water absorption; water solubility; mechanical, thermal, optical and morphological properties	Chitosan/TEO films showed higher antioxidant activity than neat chitosan; T2 film, with a chitosan: TEO ratio of 4:1, showed the best mechanical performance and reduced oxidation of fried potato fingers after 7 days at 60 °C	Not experimentally evaluated	Food validation was limited to an accelerated oxidation test on fried potato fingers; no migration/release study, sensory evaluation, biodegradation test or microbial shelf-life analysis was reported; high TEO content caused oil droplet aggregation, non-uniform morphology and poorer mechanical properties	[2]
PVA/corn starch	Watermelon rind extract (WMRE)	Fresh-cut purple cabbage; active biodegradable packaging	Antioxidant activity; antimicrobial activity; microbial count on fresh-cut cabbage; sensory/visual acceptability; tensile strength; elongation at break; WVP; soil-burial biodegradation	Addition of 10% WMRE increased tensile strength from 19.44 to 33.67 MPa and DPPH scavenging activity from 29.21% to 63.37%; PVA/ST/WMRE reduced microbial load on fresh-cut purple cabbage after 3 days at 4 °C and showed favourable consumer acceptance	Soil burial test; PVA/ST/WMRE showed 12.6% degradation after 35 days at 5 cm soil depth	Storage validation was limited to fresh-cut purple cabbage for 3 days; the antimicrobial effect of WMRE was mainly observed against *B. cereus*; migration/release, taste/odour sensory effects and longer shelf-life validation were not assessed	[69]
Chitosan	Quercetin	rapeseed oil; antioxidant active packaging for lipid oxidation-sensitive foods	Antioxidant activity; accelerated rapeseed oil storage; peroxide value; anisidine value; TOTOX index; radical scavenging activity; WVTR; OTR; water solubility; mechanical, optical, thermal and morphological properties	Quercetin increased the antioxidant activity of chitosan films and helped to reduce oxidative deterioration of rapeseed oil during 28 days of accelerated storage at 40 °C under light. Quercetin increased the antioxidant activity of films and helped to reduce oxidative deterioration of rapeseed oil during 28 days of accelerated storage at 40 °C under light	Not experimentally evaluated	Validation was limited to accelerated rapeseed oil storage; no migration/release study, sensory evaluation, biodegradation test or real commercial packaging trial was reported; quercetin increased film opacity and stiffness and reduced elongation at break	[88]
Corn starch/κ-carrageenan	Grape seed ethanol extract (GSE)	Lard; antioxidant active packaging for lipid oxidation-sensitive foods	Antioxidant activity; antibacterial activity against *E. coli* and *S. aureus*; lard oxidation test; peroxide value; WVP; tensile strength; elongation at break; colour/opacity; FTIR, SEM, XRD and DSC analyses	GSE incorporation increased antioxidant and antibacterial activity and slowed lard oxidation during 7 days of accelerated storage at 60 °C	Not experimentally evaluated	Validation was limited to accelerated lard oxidation and in vitro antibacterial tests; no migration/release study, sensory evaluation, biodegradation test or real shelf-life study on a complex food matrix was reported	[21]
Furcellaran	Silver nanoparticles	Gouda and quark cheese; antimicrobial active packaging for dairy products	Real-food cheese storage; antimicrobial effect against yeasts and moulds; total bacteria count; *Lactococcus* count; water content; water activity; pH; organoleptic quality; silver migration	Film improved the microbiological quality of cheese during storage, slowing yeast growth in Gouda and inhibiting yeast growth in quark; mould growth was also inhibited in Gouda	Not experimentally evaluated	Film negatively affected organoleptic quality after storage, mainly due to changes in appearance, smell and consistency; water loss was associated with high WVTR; silver migration into cheese was detected; biodegradation and complete toxicological safety were not directly assessed	[17]
PLA/chitosan	Nisin and EDTA	Grouper fish fillets; antimicrobial active packaging for fish preservation	Antibacterial activity against *E. coli* and *S. aureus*; fish fillet preservation; mesophilic and psychotropic bacteria; coliforms, *Aeromonas*, *Pseudomonas* and *Vibrio* counts; TVBN; pH; mechanical properties; WVTR; moisture content; overall migration in food simulants	Incorporation of nisin and EDTA improved the antibacterial activity of PLA/chitosan films; the C5EN1 film reduced microbial growth and TVBN formation in grouper fillets during storage, showing better preservation performance than PLA or chitosan-PLA films alone	Not experimentally evaluated	Food validation was limited to grouper fillets under short-term storage conditions; no sensory evaluation, biodegradation test or specific release kinetics of nisin/EDTA from the film was reported; incorporation of chitosan and EDTA/nisin reduced tensile strength and elongation at break and increased WVTR	[118]
Red chilto peel pectin	Red chilto peel polyphenolic and anthocyanin-rich extracts	Fresh salmon fillets; antioxidant active packaging for fish preservation	Antioxidant activity; salmon fillet storage; TBARS; peroxide value; free thiol groups; pH; SEM morphology; optical, mechanical, FTIR and WVP analyses	Chilto pectin films containing peel extracts reduced lipid and protein oxidation in salmon fillets during refrigerated storage at 4 °C; the polyphenol-rich extract film showed the best protection	Not experimentally evaluated	Validation focused mainly on the oxidative stability of salmon fillets; no microbial shelf-life analysis, sensory evaluation, migration/release study or biodegradation test was reported; extract addition reduced transparency and affected mechanical properties	[119]
PBAT	Siver–montmorillonite	Sea bream; antimicrobial active packaging for seafood preservation	Antibacterial activity against *E. coli*, *P. aeruginosa*, *S. aureus* and *B. subtilis*; sea bream storage; mesophilic coliforms and psychrotrophic bacteria; silver-ion release kinetics; WVP; oxygen permeability; XRD, TEM, UV–Vis and TGA analyses	Films showed controlled silver-ion release, improved barrier properties and strong antibacterial activity; 3 wt.% Ag-MMT inhibited native sea bream microflora and extended refrigerated storage up to 15 days	Not experimentally evaluated	Food validation was mainly microbiological and limited to sea bream stored at 4 °C; no formal sensory analysis, lipid/protein oxidation indices, biodegradation test or direct silver migration into fish were reported; silver release was assessed in acidified water rather than in the real fish matrix	[120]
Chitosan	Thyme oil nanoemulsion	Fresh raw beef meat inoculated with *E. coli*; antimicrobial active packaging	Antimicrobial activity against *E. coli* and *Bacillus subtilis*; agar well diffusion; agar dilution test; application on raw beef meat; droplet size and nanoemulsion stability; optical, SEM, WVP, mechanical, thermal and FTIR analyses	Chitosan films containing thyme oil nanoemulsion reduced *E. coli* growth on raw beef meat during 6 days at 4 °C	Not experimentally evaluated	Food validation was limited to inoculated raw beef meat; no full natural microbiota/shelf-life study, migration/release kinetics, formal sensory evaluation or biodegradation test was reported; TH-NE decreased tensile strength and water-vapour barrier performance	[8]
Chitosan	Magnolol	Chilled pork; antioxidant and antimicrobial active packaging	Antioxidant activity; antimicrobial activity against *P. aeruginosa*; inhibition of biofilm formation; pork preservation; pH; total viable count; sensory evaluation; WVP; moisture content; colour; ESEM; FTIR; XRD; TGA	Agnolol improved the antioxidant, antimicrobial and antibiofilm activity of chitmosan films; films containing magnolol controlled pH increase, reduced microbial growth and maintained better sensory quality of pork during 6 days at 4 °C	Not experimentally evaluated	Storage validation was limited to chilled pork for 6 days; migration/release of magnolol, biodegradation and detailed chemical spoilage/oxidation indices were not assessed; higher magnolol content changed film colour and slightly reduced thermal stability	[83]
Furcellaran	Gelatin hydrolysate, capsaicin/chitosan, AgNPs in yerba mate extract, curcumin and montmorillonite	Salmon fillets; active multilayer packaging for fish preservation	Antioxidant activity; antimicrobial activity; salmon fillet storage; total aerobic bacteria; yeasts and moulds; psychotropic bacteria; TBARS; water properties; contact angle; mechanical, thermal, SEM and AFM analyses	Multilayer films reduced microbial growth in salmon fillets during 12 days at 4 °C and extended microbiological shelf life by at least 3 days compared with the control	Not experimentally evaluated	In vitro antimicrobial tests showed no direct inhibition, probably due to slow release from inner layers; lipid oxidation was not reduced; sensory analysis, migration/release kinetics and biodegradation were not assessed	[80]
Gelatin/bacterial cellulose nanofibers	Curcumin and anthocyanin	Fresh pork; intelligent active packaging for fresh-keeping and freshness detection	Antioxidant activity; pH sensitivity; response to volatile ammonia; fresh pork storage; TVB-N; pH; hardness; springiness; film colour change; WVP; oxygen transmission; mechanical properties; SEM, AFM, FTIR and XRD	Films showed the best mechanical, antioxidant and pH-sensitive properties; during pork storage at 4 °C, they slowed total volatile basic nitrogen and pH increases, better maintained texture, and changed colour from yellow to red as freshness decreased	Not experimentally evaluated	Pork validation was limited to freshness indicators during refrigerated storage; no direct microbial enumeration, sensory evaluation, migration/release study or biodegradation test was reported; colour response needs broader validation for different food matrices	[121]
Carboxymethyl cellulose/sodium alginate	Red cabbage anthocyanin	Pork; dual-functional active and intelligent packaging for preservation and freshness monitoring	Antioxidant activity; antibacterial activity against *E. coli*, *B. subtilis* and *S. aureus*; NH_3_ colour response; pork storage; pH; TBARS; TVC; TVB-N; freshness-grade prediction by KNN; WVP; mechanical properties; opacity; colour stability; FTIR and SEM	MC/sodium alginate film containing 12% red cabbage anthocyanin showed good antibacterial activity, freshness-indicating response and stability; during pork storage, the CSR film helped maintain pork quality and enabled freshness classification, with the KNN model reaching 93.3% recognition accuracy	Not experimentally evaluated	Validation was limited to pork storage; migration/release of anthocyanins, biodegradation, sensory acceptance and consumer perception were not assessed; colour-based freshness prediction requires broader validation under different food matrices and storage conditions	[92]
Faba bean flour	Sumac extract	Chicken breast; antimicrobial and antioxidant active packaging for poultry preservation	Antioxidant activity; antimicrobial activity against *S. aureus* and *E. coli*; chicken breast storage; total bacterial count; WVP; moisture content; solubility; opacity; colour; tensile strength; elongation at break; TPC; FTIR and TGA	Films containing the highest sumac extract level showed the strongest antioxidant and antimicrobial performance; FB_S_4 inhibited *S. aureus*, improved mechanical and barrier properties, and retarded spoilage of chicken breast during refrigerated storage at 4 °C until day 7	Not experimentally evaluated	Food validation was limited to microbiological shelf-life of chicken breast; no migration/release study, sensory evaluation, lipid oxidation analysis in food or biodegradation test was reported; antimicrobial activity was effective against *S. aureus* but not against *E. coli*	[122]
Chitosan	Zinc oxide nanoparticles synthesized using apple peel extract	Fresh poultry meat; antimicrobial and antioxidant active packaging	Antimicrobial activity against *E. coli* and *S. aureus*; poultry meat storage; TMAM; TPAM; Enterobacteriaceae; TBARS; pH; TVB-N; meat colour; moisture; total zinc migration	Films reduced lipid oxidation, helped preserve the reddish colour of poultry meat and slowed microbial growth during refrigerated storage	Not experimentally evaluated	Zinc release into meat was detected, so further safety and consumer-exposure studies are needed; biodegradation, sensory acceptance and environmental fate after disposal were not directly assessed; higher ZnO levels did not consistently improve antimicrobial performance	[22]
PCL/PLA	Green tea extract	Cocktail sausage; antimicrobial and antioxidant active packaging for meat-product preservation	Antioxidant activity; antimicrobial activity against *E. coli* and *L. monocytogenes*; release in food simulants; sausage storage; total viable count; psychrophilic count; lactic acid bacteria; moulds and yeasts	Films containing 30% green tea extract showed the highest antioxidant and antimicrobial activity; when applied to cocktail sausages, films reduced microbial growth and lipid oxidation during refrigerated storage after 28 days	Not experimentally evaluated	Food validation was limited to one meat product, cocktail sausage, under refrigerated storage; no sensory evaluation, release/migration assessment in the real food matrix, or direct biodegradation test was reported.	[72]
*Alyssum homolocarpum* seed gum	Microencapsulated *Echinacea purpurea* extract (MEE)	Quail meat; antimicrobial and antioxidant edible active packaging	Antioxidant activity; antibacterial activity against *E. coli* and *S. aureus*; controlled release kinetics; quail meat storage; total bacterial count; coliform count; *Staphylococcus* count; pH; TBA/TBARS; WVP; water solubility; tensile strength; elongation; colour; FTIR, SEM and DSC	Film containing 10% microencapsulated *Echinacea purpurea* extract reduced microbial growth, limited pH increase and slowed lipid oxidation of quail meat during 14 days of refrigerated storage	Not experimentally evaluated	Release was evaluated in water rather than in the real meat matrix; validation was limited to quail meat for 14 days; sensory acceptance, migration assessment and direct biodegradation testing were not reported; higher MEE levels increased film porosity and water solubility	[123]

## Data Availability

The data presented in this study are available on request from the corresponding author.

## Abbreviations

The following abbreviations are used in this manuscript:

ABTS	2,2′-azino-bis(3-ethylbenzothiazoline-6-sulfonic acid).
AFM	Atomic force microscopy.
Ag-MMT	Silver–montmorillonite.
AHSG	Alyssum homolocarpum seed gum.
AgNPs	Silver nanoparticles.
CEO	Clove essential oil.
CFU	Colony-forming units.
CMC	Carboxymethyl cellulose.
DPPH	2,2-diphenyl-1-picrylhydrazyl.
DSC	Differential scanning calorimetry.
EAB	Elongation at break.
EDTA	Ethylenediaminetetraacetic acid.
FTIR	Fourier-transform infrared spectroscopy.
GRP	Ginseng residue polysaccharides.
GTE	Green tea extract.
HPH	High-pressure homogenization.
KNN	K-nearest neighbours.
LAB	Lactic acid bacteria.
MC	Moisture content.
MDA	Malondialdehyde.
MEE	Microencapsulated Echinacea purpurea extract.
MMT	Montmorillonite.
NPs	Nanoparticles.
OTR	Oxygen transmission rate.
PBAT	Poly(butylene adipate-co-terephthalate).
PCL	Polycaprolactone.
PE	Polyethylene.
PET	Polyethylene terephthalate.
PLA	Polylactic acid.
PP	Polypropylene.
PS	Polystyrene.
PVA	Polyvinyl alcohol.
PVC	Polyvinyl chloride.
REO	Rosa damascena essential oil.
SA	Sodium alginate.
SEM	Scanning electron microscopy.
TBA	Thiobarbituric acid.
TBARS	Thiobarbituric acid-reactive substances.
TBC	Total bacterial count.
TEO	Tulsi essential oil.
TGA	Thermogravimetric analysis.
TH-NE	Thyme oil nanoemulsion.
TPC	Total phenolic content.
TS	Tensile strength.
TVB-N	Total volatile basic nitrogen.
TVC	Total viable count.
UV–Vis	Ultraviolet–visible spectroscopy.
WMRE	Watermelon rind extract.
WS	Water solubility.
WVP	Water vapour permeability.
WVTR	Water vapour transmission rate.
XRD	X-ray diffraction.
ZnO NPs	Zinc oxide nanoparticles.
